# Preparation for the next COVID-19 wave: The European Hip Society and European Knee Associates recommendations

**DOI:** 10.1007/s00167-020-06213-z

**Published:** 2020-08-17

**Authors:** Simon T. Donell, Martin Thaler, Nicolaas C. Budhiparama, Martin A. Buttaro, Antonia F. Chen, Claudio Diaz-Ledezma, Bruce Gomberg, Michael T. Hirschmann, Theofilos Karachalios, Alexey Karpukhin, Nemandra Amir Sandiford, Hongyi Shao, Reha Tandogan, Bruno Violante, Luigi Zagra, Nanne P. Kort

**Affiliations:** 1 Norwich Medical School, University of East Anglia, Norwich, UK; 2 Department of Orthopaedic Surgery, Medical University of Innsbruck, Innsbruck, Austria; 3 Nicolaas Institute of Constructive Orthopaedic Research and Education Foundation for Arthroplasty and Sports Medicine, Jakarta, Indonesia; 4 Italian Hospital in Buenos Aires, Potosi 4247, Buenos Aires, Argentina; 5 Department of Orthopaedic Surgery, Harvard Medical School, Brigham and Women’s Hospital, Boston, MA USA; 6 Jefe Subrogante Unidad de Ortopeda y Traumatologia, Hospital El Carmen and Clinica Redsalud Santiago, Santiago, Chile; 7 OA Centers for Orthopaedics, Portland, ME USA; 8 Department of Orthopaedic Surgery and Traumatology, Kantonsspital Baselland, (Bruderholz, Liestal, Laufen), Bruderholz, 4101 Basel, Switzerland; 9 Orthopaedic Department, Faculty of Medicine, School of Health Sciences, University General Hospital of Larissa, University of Thessalia, Volos, Greece; 10 The Federal Centre of Traumatology, Orthopedics and Arthroplasty, Cheboksary, Russia; 11 Joint Reconstruction Unit, Southland Hospital, Invercargill, New Zealand; 12 Department of Joint Surgery, Beijing Jishuitan Hospital, Beijing, China; 13 Ortoklinik and Cankaya Orthopedics, Ankara, Turkey; 14 Orthopaedic Department, Istituto Clinico Sant’Ambrogio IRCCS Galeazzi, Milan, Italy; 15 Hip Department IRCCS Istituto Ortopedico Galeazzi, Milan, Italy; 16 CortoClinics, Schijndel, The Netherlands

**Keywords:** SARS-Cov-2, COVID-19, Second wave, Second phase, Hip, Knee, Arthroplasty, Recommendations

## Abstract

**Purpose:**

To plan for the continuance of elective hip and knee arthroplasty during a resurgence or new wave of COVID-19 infections.

**Method:**

A systematic review was conducted using the terms “COVID-19” or “SARS-Cov-2” and “second wave”. No relevant citations were found to inform on recommendations the plan. Therefore, an expert panel of the European Hip Society and the European Knee Associates was formed to provide the recommendations.

**Results:**

Overall, the recommendations consider three phases; review of the first wave, preparation for the next wave, and during the next wave. International and national policies will drive most of the management. The recommendations focus on the preparation phase and, in particular, the actions that the individual surgeon needs to undertake to continue with, and practice, elective arthroplasty during the next wave, as well as planning their personal and their family’s lives. The recommendations expect rigorous data collection during the next wave, so that a cycle of continuous improvement is created to take account of any future waves.

**Conclusions:**

The recommendations for planning to continue elective hip and knee arthroplasty during a new phase of the SARS-Cov-2 pandemic provide a framework to reduce the risk of a complete shutdown of elective surgery. This involves engaging with hospital managers and other specialities in the planning process. Individuals have responsibilities to themselves, their colleagues, and their families, beyond the actual delivery of elective arthroplasty.

## Introduction

### Scope

These recommendations follow those from the European Hip Society and European Knee Associates *Recommendations for Resuming Elective Hip and Knee Arthroplasty in the Setting of the COVID-19 Pandemic* [21]. The aim is to help orthopaedic surgeons across Europe (and a wider global audience) with a special interest in elective hip and knee arthroplasty prepare for the resurgence or new outbreak of COVID-19 in their locality. This involves planning resources and mitigation procedures that minimise the negative effects of the necessary restrictions imposed on their patients, their families and themselves.

### Definition of pandemic wave

The use of the term “wave” was first used in the 1889–1892 respiratory virus epidemic in Russia, which had two phases [19]. The term is inaccurate, since waves are preceded by troughs and are rhythmic in nature. The theory of second waves is based on the 1918–1920 “Spanish flu” pandemic, which may have started in a US Army camp in Kansas, a British camp in Etaples in France, or possibly German concentration camps [19]. However, the data from this outbreak are not robust; it is not even certain that the cause was an influenza virus, nor the role of bacterial superinfections. Influenza epidemics tend to be seasonal in nature. It is not known what pattern the COVID-19 will take, and whether there will be a second wave [19].

Having said that, a “wave” follows the pattern of an increase and then a decrease in numbers affected. In a pandemic, numbers of deaths lag behind the number of infected people. The wave is completed when the mortality rate returns to the background rate. The disease activity is reported using the Rt value [23]. *R* _t_ is defined as the average number of secondary cases generated by one primary case with symptom onset on day *t*. If *R* _t_ > 1, the epidemic is expanding at time *t*, whereas *R* _t_ < 1 indicates that the epidemic size is shrinking at time *t.*

Complex mathematical local models have been developed to estimate the effectiveness of testing and tracing strategies to avoid a potential second wave of the COVID-19 epidemic [2]. Close monitoring of the Rt value is essential in predicting the risk of a resurgence or second wave of a pandemic. This requires adequate, timely, and transparent reporting of the positive patients by the local health authorities. Sustained elevation of Rt value above 1 implies an increase in cases and should be considered as a marker for a resurgence of virus.

### Background

The management of the COVID-19 pandemic in the first phase, including the treatment of the patients affected, has proved very demanding. In response, health systems in most countries stopped undertaking elective surgery (both in the state and private sectors) to free up resources for managing the sickest patients [3, 8, 24, 35, 40]. The pandemic has dramatically affected arthroplasty practice all over the world [5]. Even though there were some differences due to the severity of the spread of the infection in various countries, all elective surgery stopped, at least in Europe [35]. Minor differences have been seen in the duration of the lockdown, which has lasted for a minimum of 2 months [43]. The economic impact on the health systems has been tremendous, and still to be realised. Decrease in orthopaedic elective operations has occurred globally. In USA at the beginning of 2020, almost 30,000 primary arthroplasties were being performed per week. Assuming 50% have been cancelled, then 15,000 have been postponed per week. This was seen in 2008 when the global economic recession hit the USA [15]. Orthopaedic implant companies, private healthcare centres, and healthcare professionals, have all lost an unknown amount of income. The efficiency of the system has suffered a major loss, even though there are no more official restrictions to clinical volumes [11, 31]. Due to things such as strict prevention protocols, social distancing, team disruption, reallocation of beds, and shortage of personal protective equipment, the number of cases performed per day diminished, directing impacting elective waiting times. As a consequence, we are seeing an increase of costs coupled with a reduction in efficiency, whose real effects on healthcare economic viability are still to be evaluated. In this scenario, surgeons must still offer the best treatment for their patient whilst navigating a possible resurgence or second wave [6].

Most European countries have moved to a post-first wave period. A resurgence or a second wave of COVID-19, regardless of whether there are stringent or relaxed policies for mitigation, will then have a further severe impact [20, 36]. Adult hip and knee elective reconstructive surgery has restarted so as to avoid the serious effects of delaying surgery on patients’ symptoms, function, and quality of life [27, 28]. Scientific bodies in both Europe and North America have developed the guidelines for a progressive restart, paying attention to the safety of patients and staff [21]. However, the scientific basis that underpins the guidelines is poor, despite the influx of COVID-19 articles, most of dubious quality. As many of the countries begin to think about reducing restrictions to allow social an economic recuperation, the World Health Organisation (WHO) revealed that highest daily incidence of confirmed cases occurred on 22 June 2020 [22]. The numbers of cases of SARS CoV-2 are also increasing daily in South America, North America, Africa, and India [22]. Overall, the number of cases continues to increase [19]. Detection of new cases in Beijing has led to a renewed lockdown there. The potential for a second wave of infection has been highlighted in both the medical and non medical literature [41]. Potential reasons for this include:

- Relaxing the social distancing and population behaviour measures before adequate suppression of local cases from failure to detect all cases in a specific population.
- Introduction of new cases via travel or interaction with infected individuals who have not been screened.
- Initial failure to report or test populations at risk during the initial wave, e.g., in nursing homes [16].
- Failure of the population to acquire herd immunity. In most countries, the infection rate is less than 4% [13, 22].

Several of these factors, either in isolation or combined, can result in a resurgence of cases. Further waves are possible in the future. Whilst the timing and location of a potential second wave is difficult to predict, valuable lessons have been learned from the first wave, which can inform on the management of a resurgence or a subsequent wave.

Lockdown has been effective in reducing transmission of the virus. It has been estimated to have prevented or reduced 530 million infections in several countries [18]. Screening and contact tracing and early detection have significantly reduced transmission rates [32]. Isolation and quarantine measures have reduced community and national spread, especially amongst at-risk groups (elderly, comorbidities, lower socio-economic, and certain ethnic groups). The use of personal protective equipment (PPE), by those at risk of exposure, has reduced the incidence of COVID-19 spread in healthcare and other key workers [25]. In this respect, the definition of ‘key worker’ has to be clear as, for businesses, healthcare institutions, and society as a whole to function, a variety of individual skills from cleaners to surgeons are required. A vaccine is being discussed, but this has not yet been developed.

The important difference between applying these measures to a second outbreak will be to ameliorate the effects of the isolation of people, with its impact on their physical and mental health (in particular, populations at risk, and those in full-time education) and the reduction in economic activity. It is possible that ‘normal life’ will be adapted to have several of these intervention measures, including the daily routine.

The COVID-19 pandemic appeared suddenly and found the global medical community relatively unprepared despite the past experience of the spread of other viral infections. It is inevitable that a resurgence or new wave of CODID-19 infections will have a further severe impact on elective arthroplasty services when restrictions are imposed on a locality. This paper reports the recommendations for individual surgeons on how to prepare for a new lockdown to minimise this impact on their elective hip and knee arthroplasty practice.

## Materials and methods

The European Hip Society (EHS) and the European Knee Association (EKA) formed a panel of experts to review the literature and to come to a consensus on the recommendations.

### Systematic review

A systematic review of the available literature was performed up to the 3rd July 2020 using the key words “COVID-19” or “SARS-Cov-2” and “second wave”. The following databases were accessed: PubMed, Scopus, and Google Scholar. All available articles were accessed, and there was no language exclusion. SD and TK conducted the search. NC acted as the arbiter if there was any disagreement.

A total of 61 citations were found of which 11 were letters or comments’ warning of specific problems with a second wave and advising on getting prepared, three were about avoiding a second wave, and the rest had no relevance to preparation for a new wave, and none gave any details on recommendations. Within these 61, the two orthopaedic-related citations were principally concerned with the current management [12, 37]. Included was an open letter in the British Medical Journal to all the UK political parties’ leaders, from a group of senior UK medical leaders. They concluded that rapid attention was needed in a number of policy areas [1]:

- Governance including parliamentary scrutiny, and involvement of regional and local structures and leaders.
- Procurement of goods and services.
- Coordination of existing structures to optimise effective public health and communicable disease control infrastructure, resilience of the health service, and shielding of vulnerable individuals and communities.
- The disproportionate burden on black, Asian, and ethnic minority individuals and communities.
- International collaboration.

### Consensus

It was clear from the literature review that any recommendations would be based on expert opinion without any robust independent evidence to support them.

## Results

The current pandemic is unique, since there are no post-pandemic health care delivery system recovery studies, nor any theoretical studies of post-pandemic health care delivery system recovery operations [39]. However, the healthcare systems are more prepared for a second wave, with improved logistics, experienced personnel, and treatment algorithms. However, as is clear from the open letter by Adebowale et al. [1] international, national, and local policies will drive the management of a resurgence or new wave for which an individual orthopaedic surgeon has no direct responsibility.

Looking specifically at arthroplasty, most countries who have resumed elective cases have created separate pathways for medically necessary COVID-negative operations. This has been in the form of designated COVID hospitals, or physically separating patients, wards, operating theatres, and personnel, coupled with increased vigilance in disinfection measures and identifying virus carriers. The allocation of resources and hospital beds to elective procedures will be titrated against the possible need for these resources if a resurgence of the pandemic occurs. This allocation will follow the guidance of local health authorities, international health organisations, and scientific societies.

Present and future economic limitations should not diminish the advocacy for high-quality healthcare, nor should it change the ideal indications for arthroplasty. The challenge is to provide the same standard of quality with fewer resources. A second wave will put a major stress on the system. Efforts at mitigation need to be considered and put in place now, so as to maintain a high a rate of elective arthroplasty as possible, despite the strain of COVID-19 on the system. The possibility of a new slowdown of elective surgery needs to be discussed with the patients, in particular in the most severe ones where delay will lead to a worse outcome. The goal is to avoid another complete lockdown of arthroplasty surgery, even if healthcare professionals must be ready for the worst from a both clinical and economic points-of-view.

The disease will not be controlled fully until an effective vaccine has been found, or herd immunity in the population has occurred. This does not seem to be possible soon. Post-disaster scenarios for healthcare management also point out a different second wave; the surge in demand for healthcare in chronic patients who have delayed or have been unable access treatment during the disaster [33].

**Fig. 1:**
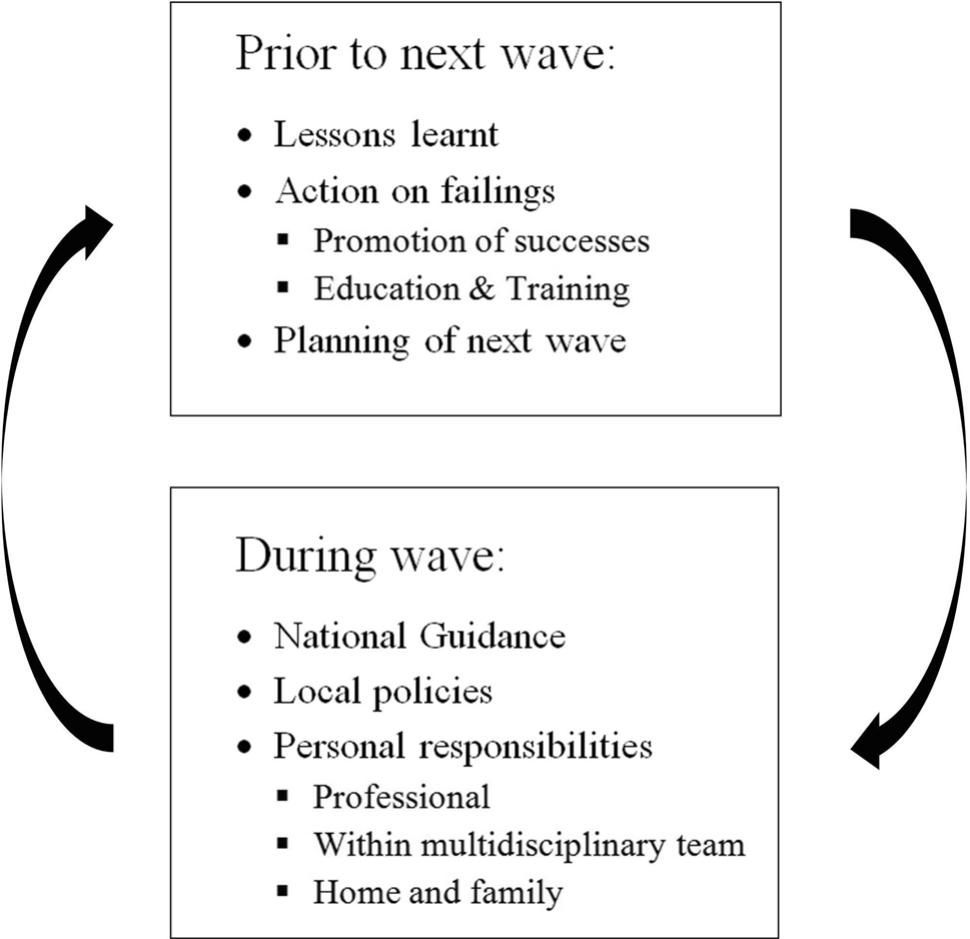
The necessary actions for planning for the next wave

Arthroplasty surgeons need to be engaged in the following:

- Prioritisation,
  Limited amount of selected arthroplasty operations have been performed during the pandemic, depending on the type of procedure, status of the patient, and regional rules of patient prioritisation. Arthroplasty for periprosthetic and femoral neck fractures were still performed, in 87% and 85% of suitable patients, respectively. However, 96% of aseptic arthroplasty revisions and 94% of elective primary total joint arthroplasty were postponed [35]. Indefinitely delaying all elective arthroplasty procedures is unacceptable. Although prioritising patients, who are low risk for COVID-19 disease transmission and postoperative complications, can be done, ethical aspects also have to be considered. Older, more vulnerable patients will suffer most. The beneficial impact of total joint arthroplasty regarding mobility, social life, work capability, prevention of cardiovascular diseases, general health, patient satisfaction, decreasing pain, and increasing joint function, is clearly recognised [35]. Postponing total joint arthroplasty leads to an increase in the use of medication and more unsatisfactory overall outcome. The prolonged time of pain and social isolation, because of immobilisation, risks their mental health. If patients have limited access to total joint arthroplasty, or have to wait for an extended period on waiting lists, the direct and indirect costs of a nation’s society will increase [34].
- Patient information,
  The individualised guidance that patients receive is the key to when deciding to undergo elective joint replacement [9]. Direct, succinct, and transparent information is recommended for communication through the coronavirus crisis [4]. It is our duty as surgeons to be committed, use shared-decision making tools [7], and back up our recommendations with up-to-date scientific information supported by scientific societies and local governments.
  Patients receive information from various sources, especially Internet sites, which are not necessarily trustworthy [26]. Even though the peak of COVID cases is declining, a recent study by Chang et al. [10] found that only 56% of the patients interviewed who were awaiting elective arthroplasty, agreed to undergo surgery. Patients waiting for knee arthroplasty were more reticent than those for hip arthroplasty. It is remarkable that none of the patients who were waiting for a revision arthroplasty agreed to undergo an operation during this period.
  Official information for patients, using an appropriate level for their clear understanding, should be available on institutional and orthopaedic societies' webpages [38]. A patient's refusal to undergo elective surgery during this time should not be synonymous with declining a joint replacement. The surgeon should consider reoffering the surgical option after a reasonable time, ideally in a scenario with less risk of coronavirus transmission.
- Legal aspects,
  Orthopaedic surgeons should be aware of the presence of several new legal issues during the COVID-19 pandemic. There is a risk for claims against healthcare facilities, and, therefore, orthopaedic surgeon by association. The claims could be for:
  1. Failing to prepare and respond appropriately to a COVID-positive patient.
  2. Fail to diagnose a COVID-19 patient in a timely fashion and proper fashion.
  3. Negligence in taking appropriate precautions to prevent or limit exposure and spread of the virus.
  4. Negligence in treating a COVID-positive patient in a timely and proper fashion.
  Therefore, it is incumbent on the surgeon to be familiar with regional standards of care, in addition to national, state, local, and hospital protocols, for the medical and surgical treatment during the COVID-19 pandemic. Although there is often implied immunity given to treating physicians during the pandemic, these are usually reserved for “front line” staff. Orthopaedic surgeons may not be viewed as such. It is important to understand the patient’s legal rights during this time.
- Consent
  Appropriate information must be accompanied by a specifically designed consent form, which should be produced at the centre where the surgery is to be performed. Patients must receive the message that elective procedures will be carried out to the highest technical standards whilst maintaining preventative measurements. The eventual modifications in peri-operative protocols (early hospital discharge, telemedicine, tele-rehabilitation, etc.) are intended to provide the best possible outcome. Notwithstanding, patients should comprehend and accept that the occurrence of adverse events and complications can be minimised but not abolished.
  Patients should understand that, in the event of an official declaration of a resurgence, or further wave, of COVID cases in a given area, the scheduling of elective cases may be altered and even cancelled. In countries with a waiting list for joint replacement, patients should be informed that of the expected prioritisation decisions and how this will impact on their own waiting time. Recommendations to prioritise, for instance younger individuals with a few comorbidities, could generate a delay in the surgical access of those with greater peri-operative risk. Therefore, the possibility of a less successful functional result in those patients is a possibility [44].
  Best practice is to document informed consent carefully. Although there may be implied relief from documentation during the pandemic, the benefits of detailed documentation outweigh any intended relief. Documentation is the single best way to protect the surgeon and the patient. The surgeon–patient discussion preceding arthroplasty should be documented; especially the changes from previous practice because of the COVID-19 pandemic. Several national societies have publicised a need for a specific, informed consent. At the very least, the following should be documented:
  1. COVID-19 has been declared a pandemic by the World Health Organisation.
  2. The surgeon and facility closely monitor the local situation and put in place reasonable measures aimed to reduce the spread of the virus.
  3. The patient understands that arthroplasty, except for impending or catastrophic failure, periprosthetic fracture, and infection treatment, is an elective procedure.
  4. The patient acknowledges that, although they may have a negative symptom profile or coronavirus negative test, they still may have a COVID-19 infection. Should they develop COVID-19 infection they have an increased risk of significant complications and death.
  5. The patient acknowledges the option to delay surgery further was provided, and that the delay may lead to a worse overall outcome [42, 45].
- The role of orthopaedic surgeons,
  During the next wave of the COVID-19 pandemic, surgeons will have personal responsibilities. The actions necessary need to be planned for now (Fig. 1). There will be National Guidance and local policies that need to be adhered to including around COVID-19 screening for patients and staff, but surgeons themselves have obligations that must be considered and actioned during the phase between waves.

There are professional responsibilities [14] to oneself, to the multidisciplinary team, and to one’s family. A period of reflection on what went well and what could have been done better during the first wave and the lockdown needs to be considered by oneself, the multidisciplinary team, and one’s family. Included in this is both the physical and mental wellbeing of all those involved, and any special requirements on individuals in the group from their personal risks factors, especially those with a black, Asian, or minority ethnic background [29]. It should include the type and availability of personal protective equipment, how to manage if sickness strikes, and such mundane things as how to get one’s hair cut.

On a personal level, the surgeon should consider what professional training they require, particularly if rostered into a new role from elective arthroplasty, e.g., providing cover for trauma. More training on managing ethical problems has been highlighted [30], although the legal ramifications may depend on the country of one’s practice [17]. One should also consider how to manage personal bereavement and its legal manifestations, including writing a will.

The recommendations are reported in Table 1.

**Table 1:** The recommendations of the EHS–EKA for planning for the next phase of the SARS-Cov-2 pandemic

Review previous wave
i. Note what went well
ii. Create standard operating procedures (SOP) where appropriate
iii. Create hospital and departmental guidelines
iv. Consider what needs improving
v. Undertake literature review to define best practice
vi. Create guidelines and SOPs for next wave
Prepare for next wave
i. Personal
a. Professional
Consider any necessary actions required to minimise the risk of adverse outcomes should you become COVID-19 positive
Reflect on the previous wave and arrange any necessary professional training needed, including managing ethical dilemmas, e.g., patient prioritisation for elective arthroplasty
Stay up-to-date with guidelines and SOPs for COVID-19
Be involved in department-level activities to create the guidance and SOPs
b. Multidisciplinary
Consider the relevant support needed for members of the multidisciplinary team, especially members who are at risk of an adverse outcome if they get a COVID-19 infection
c. Family
Consider how to manage personal bereavement
Write a will
Get personal and family finances in order and plan how to cope with a drop in income
Plan for home education of children
Plan social activities for the family. Check computer systems and internet connections. Train family members in use of social media to stay in touch
Consider how food supply is obtained during next wave
Consider how medical services are accessible, and medication obtained. Be clear about routes to help if sickness occurs within the family
Plan how to manage to get hair cut
ii. Departmental
a. Use local governance meetings to obtain departmental consensus
b. Involve other relevant disciplines in guidance and SOP production
c. Use governance and department meetings to inform colleagues of the literature and science of managing COVID-19, the new SOPs and the guidelines
d. Department leads should engage with hospital management on the deployment of the department during the next wave, and the necessary training required for members of the orthopaedic department. Where appropriate these can be included in mandatory training
e. Create a policy to maintain elective arthroplasty during the next wave, with allocation of the necessary resources to achieve this
iii. Hospital/healthcare facility
a. Incorporate local guidance into departmental guidelines
iv. National
a. Incorporate national policy and guidance into departmental guidelines
During the next wave
i. Collect data on performance
ii. Audit use of guidance and guidance knowledge
iii. Be transparent and non-confrontational over problems and data
iv. Obtain feedback from staff at regular review meetings
v. Work with management to maximise all the patients’ outcomes
vi. Support colleagues in difficulty

## Discussion

The recommendations provide a framework within which data collection and analysis are the key for the planning and refining of the response to a new phase of SARS-Cov-2. Further phases are expected, and new infective organisms with a global dissemination are expected. This means that the actual details of managing an epidemic at a local level will be changing over time as the resources needed to manage a particular outbreak will differ. This difference may be national or regional, and so, details cannot be assume to apply universally.

Although the details of a hospital’s preparation for the next wave in maintaining elective primary arthroplasty are tied to national guidance, much can be done by an individual to mitigate on the problems that can arise at a personal and family level. It is here that action should be taken to relieve the burden and stress of a lockdown.

## Conclusion

The preparation for the next wave starts with reviewing the previous wave. Between the waves preparation is needed at a personal, departmental, and hospital level and national levels. At a personal level, there are professional activities that need to be pursued, as well as to the multidisciplinary team and to ones family. During the next wave, accurate data collection with transparent dissemination is essential and needs to be planned for at this stage.
